# Therapeutic Potential of Combining IL-6 and TNF Blockade in a Mouse Model of Allergic Asthma

**DOI:** 10.3390/ijms23073521

**Published:** 2022-03-24

**Authors:** Olga A. Namakanova, Ekaterina A. Gorshkova, Ruslan V. Zvartsev, Sergei A. Nedospasov, Marina S. Drutskaya, Ekaterina O. Gubernatorova

**Affiliations:** 1 Engelhardt Institute of Molecular Biology, Russian Academy of Sciences, 119991 Moscow, Russia; gorshsama@gmail.com (E.A.G.); zvartsev@eimb.ru (R.V.Z.); marinadru@gmail.com (M.S.D.); 2 Department of Immunology, Faculty of Biology, Lomonosov Moscow State University, 119234 Moscow, Russia; 3 Division of Immunobiology and Biomedicine, Center of Genetic and Life Sciences, Sirius University of Science and Technology, 354340 Federal Territory Sirius, Russia

**Keywords:** HDM-induced asthma, anti-cytokine therapy, Th2-induced eosinophilia, Th17/Th1-induced neutrophilia

## Abstract

Combined anti-cytokine therapy is a promising therapeutic approach for uncontrolled steroid-resistant asthma. In this regard, simultaneous blockade of IL-4 and IL-13 signaling by Dupilumab (anti-IL-4Ra monoclonal antibody) was recently approved for severe eosinophilic asthma. However, no therapeutic options for neutrophilic asthma are currently available. Recent advances in our understanding of asthma pathogenesis suggest that both IL-6 and TNF may represent potential targets for treatment of severe neutrophilic asthma. Nevertheless, the efficacy of simultaneous pharmacological inhibition of TNF and IL-6 in asthma was not yet studied. To evaluate the potency of combined cytokine inhibition, we simultaneously administrated IL-6 and TNF inhibitors to BALB/c mice with HDM-induced asthma. Combined IL-6/TNF inhibition, but not individual blockade of these two cytokines, led to complex anti-inflammatory effects including reduced Th2-induced eosinophilia and less prominent Th17/Th1-mediated neutrophilic infiltrate in the airways. Taken together, our results provide evidence for therapeutic potential of combined IL-6/TNF inhibition in severe steroid-resistant asthma.

## 1. Introduction

Allergic asthma is a chronic progressive inflammatory disease affecting millions of people worldwide. The typical asthma symptoms include wheezing, breathlessness, and chest tightness, which exacerbate following allergen exposure and may eventually cause secondary complications. Despite the success of anti-inflammatory steroid therapy, this approach remains symptomatic and is not always effective, especially, in the case of such asthma endotypes as severe eosinophilic and neutrophilic asthma.

Allergic asthma is associated with the sensitization to some common aeroallergens, such as house dust mite, pollens, fungi, and animal dander [1]. Repetitive exposure of the airways to these allergens leads to the activation of antigen-presenting cells, especially, macrophages. Expansion of T-helper populations, either Th2 or Th1/Th17, that secrete cytokines, promote granulocyte infiltration in the airways with subsequent release of pro-inflammatory factors [2]. The type of inflammatory response varies in different patients and may be predominantly Th2- or Th17-mediated, as well as the full spectrum of mixed Th2/Th17 immune responses [3]. All of these asthma subtypes may result in severe uncontrolled inflammation. However, patients that displayed Th17-mediated inflammation have a higher likelihood of uncontrolled asthma manifestation due to neutrophil recruitment [4]. Standard steroid-based treatment of neutrophilic response is not effective; steroids inhibit neutrophil apoptosis and therefore enhance neutrophil-mediated inflammation [5,6]. Thus, the development of novel therapeutic strategies with tight regulation of a particular arm of the immune response in asthma remains an extremely important task.

Dissecting the molecular cascades of asthma pathogenesis in search for effective therapy supports the idea of a personalized approach that considers different disease-driving mechanisms. Cytokines in asthma are implicated both in detrimental and protective pathways. Recent studies demonstrated that the inhibition of selected cytokines may be helpful in controlling moderate and severe asthma [7,8,9]. TNF and IL-6 are the two cytokines with a broad spectrum of immunodulatory effects, yet in the context of asthma they both can be defined as pathogenic. TNF represents an important biomarker in severe asthma and TNF gene polymorphisms are associated with increased risk of asthma development [10]. TNF is also essential for leukocyte recruitment to the inflamed lungs [11,12], leukotriene production [13], and for TNFR1-dependent smooth muscle contraction leading to bronchoconstriction [14,15]. Furthermore, TNF is crucial for the inflammatory imprinting of airway macrophages in response to allergen challenge [16]. Unfortunately, anti-TNF therapy failed to provide significant protective effect in clinical trials [17], even though no complications were reported [18,19]. IL-6, on the other hand, orchestrates differentiation and commitment of different T-cell subsets, especially Th17-cells [20]. Th17-cells are the gatekeepers of mucosal homeostasis and are involved in neutrophil recruitment, thus, contributing to severe asthma [21,22]. Limited data are available on the types of asthma-associated immune responses under IL-6 blockade [23]. To deepen our understanding of possible functional interaction between TNF and IL-6 in the context of allergic asthma, in the present study using a mouse model of HDM-induced asthma we examined the effects of combined IL-6 and TNF inhibition.

## 2. Results

### 2.1. Combined Inhibition of IL-6 and TNF in Mice with Acute HDM-Induced Asthma Results in Decreased Granulocyte Infiltrate in the Airways

**Figure 1:**
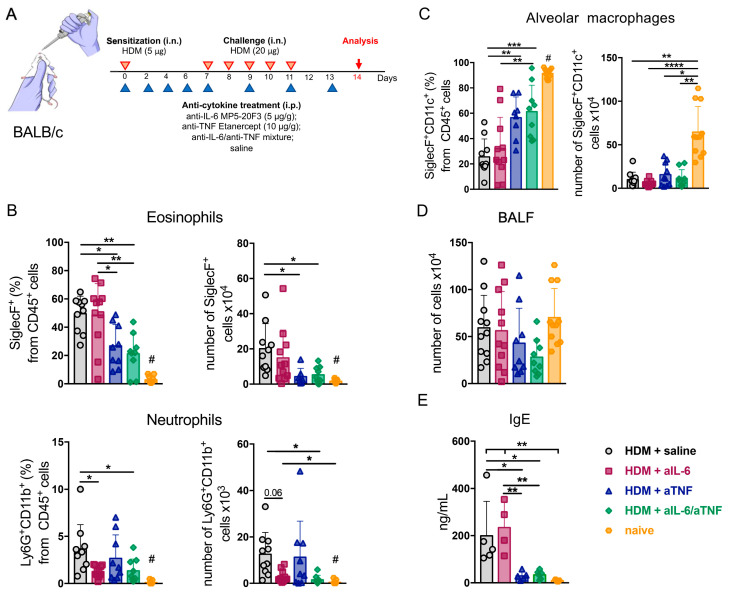
Combined pharmacological inhibition of TNF and IL-6 in acute HDM-induced asthma. (**A**) Scheme of the experiment. Acute asthma was induced in 6–8-week old BALB/c mice by daily i.n. administration of 20 µg HDM extract per mouse for six days with the sensitization of 5 µg HDM one week prior to the main course (red arrows). Anti-IL-6 antibody (MP5-20F3) (5 µg/g of body weight), anti-TNF inhibitor (Etanercept) (10 µg/g of body weight), and saline as a control were administered i.p. every 48 h for 13 days according to the scheme (blue arrows). Prior to i.n. administration of HDM, mice were anesthetized by 3% isoflurane delivered with oxygen. BALF was collected for analysis 48 h after the last challenge. Frequencies (%) of eosinophils (Siglec-F^+^ CD11c^−^), neutrophils (Ly6G^+^ CD11b^+^) (**B**) and alveolar macrophages (SiglecF^+^ CD11c^+^) (**C**) gated on CD45^+^ live cells in the BALF were assessed by flow cytometry. (**D**) Total cell numbers in the BALF. (**E**) IgE production (ng/mL) in the BALF was determined by ELISA on day 14. Each point on a diagram represents a single mouse (5–12 mice in each group); mean ± SD. * *p* < 0.05; ** *p* < 0.01; *** *p* < 0.001; **** *p* < 0.0001; #—**** *p* < 0.0001 (naïve vs. HDM + saline), *** *p* < 0.001 (naïve vs. HDM + aIL-6), ** *p* < 0.01 (naïve vs. HDM + aTNF); * *p* < 0.05 (naïve vs. HDM + aIL-6/aTNF) (one-way ANOVA test was used). HDM, house dust mite; BALF, bronchoalveolar lavage fluid.

Combined anti-cytokine therapy is attracting interest as a way to overcome multiple detrimental effects of pro-inflammatory cytokines [8]. In the current study, we hypothesized that combined IL-6/TNF inhibition may be beneficial in the context of severe asthma. To this end, we induced acute asthma in BALB/c mice by daily administration (i.n.) of 20 µg HDM per mouse for six days with the sensitization of 5 µg HDM one week prior to the main course, as shown schematically in Figure 1A. Anti-IL-6 antibodies (MP5-20F3) (5 µg/g of body weight) or TNF inhibitor (Etanercept) (10 µg/g of body weight), or the mixture of these two inhibitors and saline as a control were administered intraperitoneally every 48 h for 13 days (Figure 1A). The total cell number in the BALF did not differ in either monotherapy or combined cytokine inhibition groups (Figure 1D). At the same time, combined TNF and IL-6 ablation resulted in a significant reduction in granulocyte counts in the BALF (Figure 1B). Interestingly, mice receiving anti-IL-6 had decreased frequency and number of neutrophils, but not eosinophils, whereas TNF blockade predominantly suppressed accumulation of eosinophils (Figure 1B). Moreover, anti-TNF alone as well as simultaneous inhibition of both TNF and IL-6 were able to support the maintenance of alveolar macrophage population as compared to the control group and to mice with pharmacological IL-6 ablation (Figure 1C). Alveolar macrophages are known as the main subset of immune cells in steady-state lungs that controls inflammation (Figure 1C) [24]. We also found that combined IL-6/TNF blockade resulted in the marked decline in IgE levels in the BALF as compared with the control group (Figure 1E). IgE is a crucial factor in Th2-mediated immunity and atopy [25]. Interestingly, mice with TNF blockade showed the same effect, in contrast to anti-IL-6 monotherapy (Figure 1E), indicating that IgE levels decreased due to TNF inhibition.

These observations suggest that simultaneous administration of anti-IL-6 and anti-TNF more effectively extinguishes the onset of acute asthma by functionally suppressing both eosinophils and neutrophils in the airways.

### 2.2. Simultaneous Ablation of IL-6 and TNF Significantly Reduced Th2- and Th1-Mediated Inflammation in the Lungs

**Figure 2:**
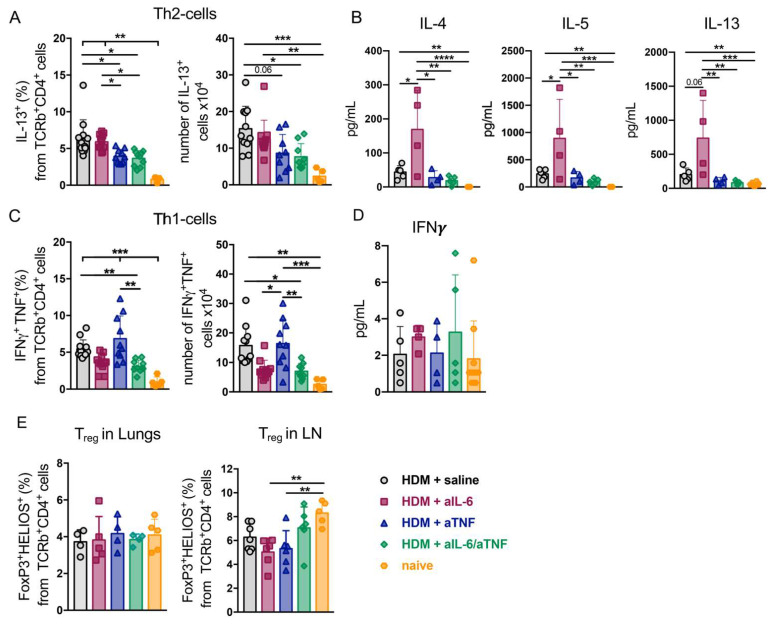
Th2- and Th1-cell inflammatory responses in the lungs of HDM-treated mice with simultaneous administration of IL-6 and TNF inhibitors. (**A**) Frequency (%) of Th2-cells (IL-13^+^) among TCRb^+^ CD4^+^ cells in the lungs. (**B**) Protein levels of Th2-associated cytokines (pg/mL) in the BALF were measured by multiplex analysis on day 14. (**C**) Frequency (%) of Th1-cells (TNF^+^ IFNγ^+^) gated on TCRb^+^ CD4^+^ live cells in the lungs. (**D**) IFNγ production (pg/mL) in the BALF was determined by multiplex analysis on day 14. (**E**) Frequency (%) of Treg cells (FoxP3^+^ HELIOS^+^) in the lungs and lymph nodes (LN). Data represent mean ± SD, 4–12 mice per group with each point representing a single mouse. * *p* < 0.05; ** *p* < 0.01; *** *p* < 0.001; **** *p* < 0.0001 (one-way ANOVA test was used).

A number of studies suggested a central role of the predominant type of T-cell response in consideration of therapeutic strategy for asthma [26]. Thus, mild asthma is characterized by Th2-cell activation, whereas severe asthma is associated with Th17/Th1-cell responses in the airways. In order to identify a particular type of T-cell response under pharmacological cytokine inhibition, FACS analysis of lung Th-lymphocyte subsets was performed (Figure S2). Administration of IL-6 inhibitor in mice with HDM-induced asthma did not result in reduced frequency of Th1-cells and IFNγ secretion (Figure 2C,D) and also the frequency and number of Th2-cells (Figure 2A) in the airways as compared to the saline control group.

Furthermore, pharmacological inhibition of IL-6 during airway inflammation was associated with a marked local increase in protein levels of IL-13, IL-4, and IL-5, which are critically involved in Th2-mediated eosinophilia (Figure 2B). Importantly, combined pharmacological inhibition of TNF and IL-6 prevented the recruitment of both Th1- and Th2-cells to the lungs (Figure 2A,C). Next, we determined whether the simultaneous blockade of TNF and IL-6 affected the accumulation of T-lymphocytes and granulocytes in the periphery. Neither granulocyte infiltration (Figure S3A) nor the composition of Th-cells significantly differed in spleens of mice with combined IL-6/TNF ablation as compared to other groups (Figure S3B). However, the frequency of Th1-cells declined in the spleens in mice with simultaneous IL-6/TNF blockade (Figure S3B). At the same time, combined anti-cytokine therapy did not affect Treg cell numbers and presumably did not contribute to suppressive environment either locally, in the lungs, or in the periphery (Figure 2E). Altogether, we concluded that combined inhibition of TNF and IL-6 more effectively reduces the inflammatory response in the lungs as compared to neutralization of these cytokines individually in acute HDM-induced mouse model of asthma.

### 2.3. Unlike Anti-TNF Monotherapy, Anti-IL-6 Treatment and Combined Pharmacological Inhibition of TNF and IL-6 Suppressed Th17-Response in the Lungs

**Figure 3:**
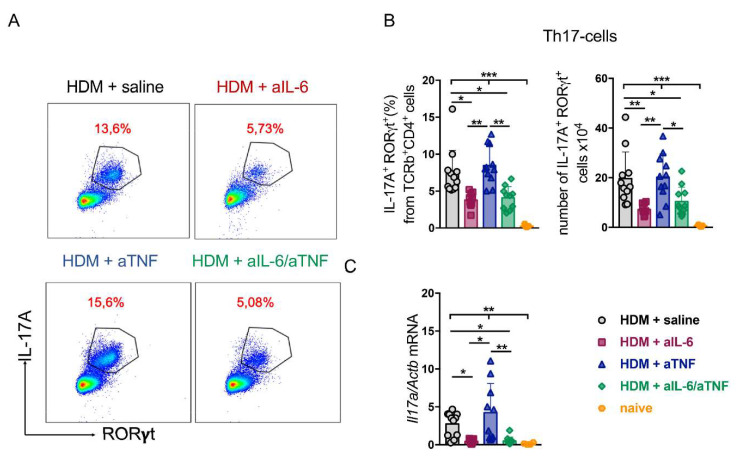
Th17-cell response in HDM-treated mice with combined anti-TNF/IL-6 treatment. Representative FACS plots (**A**) and frequency (%) (**B**) of IL-17A^+^ RORγt^+^ cells gated on TCRb^+^ CD4^+^ live cells in the lungs of control mice and mice with anti-cytokine treatment. (**C**) Relative expression of *Il17a* gene normalized to *Actb* by quantitative RT-PCR analysis in the lungs 48 h after last HDM challenge. Each point in a diagram represents a single mouse (5–12 mice in each group); mean ± SD. One-way ANOVA test revealed: * *p* < 0.05; ** *p* < 0.01; *** *p* < 0.001.

It is known that severe neutrophilic asthma is associated with activation of not only Th1-, but also of Th17-dependent pathways [27,28]. To evaluate the effects of simultaneous administration of TNF and IL-6 inhibitors, we measured the number of Th17-cells in the lungs of mice with HDM-induced asthma (Figure 3A).

We noted a significant reduction in the number and frequency of Th17-cells under combined IL-6/TNF ablation as compared to the control group (Figure 3B). Interestingly, Th17-cell population was expanded in mice with pharmacological TNF inhibition, but not in mice with simultaneous ablation of the two cytokines. Moreover, administration of anti-IL-6 antibodies only led to a significant decrease in Th17-cells in the lungs, which was in agreement with reduced neutrophil counts (Figure 1B and Figure 3B). Altogether, these data indicate that administration of anti-IL-6 agent primarily leads to suppression of Th17 response, while TNF blockade apparently contributes to Th17 accumulation (Figure 3B). We also found that the combined pharmacological inhibition of TNF and IL-6 prevented the upregulation of pathogenic *Il17a,* known to play an important role in the formation of neutrophilic asthma, whereas TNF neutralization maintained a high expression level of *Il17a* (Figure 3C). Reduction in expression levels of other asthma-associated genes was not statistically significant among all groups tested (Figure S4). In summary, these findings demonstrate the efficacy of combined pharmacological inhibition of IL-6 and TNF in suppression of Th17-mediated airway inflammation.

### 2.4. Simultaneous TNF/IL-6 Inhibition Prevented Lung Tissue Remodeling in Severe HDM-Induced Asthma

**Figure 4:**
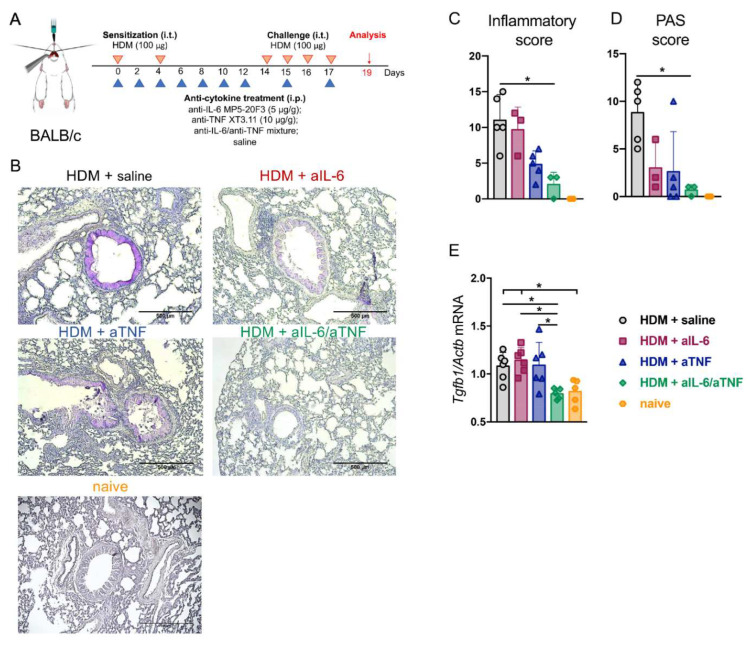
Assessment of lung histology in mice with severe HDM-induced asthma undergoing combined administration of IL-6 and TNF inhibitors. Simultaneous pharmacological inhibition of TNF and IL-6 led to diminished lung tissue remodeling in severe HDM-induced asthma. (**A**) Scheme of severe asthma model; 6–8-week-old BALB/c mice were sensitized i.t. with HDM extract on days 0 and 4 (100 μg) (red arrows). On days 14–17, mice were challenged i.t. with HDM (100 μg) (red arrows). Prior to i.t. administration of the allergen, mice were anesthetized by i.p. injection of the Zoletil/Xyla cocktail for anesthesia. Anti-IL-6 (MP5-20F3) (5 μg/g of body weight), anti-TNF (XT3.11) (10 μg/g of body weight) antibodies, and saline as a control were administered intraperitoneally prior to each immunization every 48 h for 17 days (blue arrows); 48 h after the last challenge lungs were perfused by cardiac puncture using 0.9% NaCl and fixed in 4% PFA for further histological assessment of lung tissue. (**B**) Representative PAS-stained lung tissue sections (original magnification: 50×, scale bar: 500 μm) in control mice with saline and mice injected with anti-TNF and/or anti-IL-6 antibodies (i.p.). (**C**) Lung inflammation and (**D**) PAS-positive cell score. (**E**) Relative expression of *Tgfb1* gene normalized to *Actb* by quantitative RT-PCR analysis in the lungs 48 h after last HDM challenge. Data represent mean ± SD, 5–6 mice per group. * *p* < 0.05 (one-way ANOVA test was used).

To address the functional significance of combined cytokine blockade in airway remodeling, mice were subjected to high dose of HDM for severe asthma induction [29]. Severe asthma was induced by intratracheal injection of 100 μg HDM extract on days 14–17 with the sensitization of 100 μg HDM on days 0 and 4. Anti-IL-6 antibodies (MP5-20F3) (5 μg/g of body weight) and anti-TNF antibodies (XT3.11) (10 μg/g of body weight), or a mixture of inhibitors and saline as a control were administered intraperitoneally prior to each immunization every 48 h for 17 days (Figure 4A).

Lungs were perfused by cardiac puncture using 0.9% NaCl and fixed in 4% PFA for histological analysis. Hematoxylin and PAS staining revealed reduced inflammatory cell infiltration and goblet cell population in mice with combined inhibition of TNF and IL-6 as compared to other groups (Figure 4B). These findings were corroborated by semiquantitative scoring of PAS-positive cells and of inflammatory cell infiltrate (Figure 4C,D). TGF-β is known for its capability to induce collagen production in lung tissue that may lead to fibrosis. Indeed, TGF-β expression was elevated both in animal models of lung fibrosis and in human lungs with fibrosis. Additionally, fibroblast proliferation and trans-differentiation depend on TNF- and IL-6-induced expression of TGF-β [30,31]. We found that simultaneous IL-6/TNF inhibition downregulated *Tgfb1* expression, which was not observed in mice with neutralization of these cytokines individually (Figure 4E). Nevertheless, the expression levels of *Areg* and *Col1a1*, other fibrosis-associated markers, did not significantly differ from the control group (Figure S4). These results suggest that combined pharmacological inhibition of TNF and IL-6 in the context of severe HDM-induced asthma may provide benefit in TGF-β-dependent lung remodeling, as compared to inhibition of these cytokines individually.

## 3. Discussion

Understanding critical immune processes involved in asthma pathogenesis and the role of cytokine cross-talk is important when novel therapeutic strategies targeting pathological mechanisms of disease progression are designed [26]. Owing to heterogeneity of the asthma, it is extremely difficult to identify the initial cause of the inflammatory process in each specific case, and to select an effective therapy [32]. Severe asthma, characterized by frequent exacerbations, decline in lung functions, resistance to corticosteroids, and the presence of neutrophilic infiltration in the lungs [33,34] is poorly controlled by standard treatments. Anti-cytokine therapy targeting the key pro-inflammatory mediators is highly effective in some cases of severe asthma [35]. In particular, two anti-IL-5 antibodies, Mepolizumab [36] and Reslizumab [37], and anti-IL-5R antibody, Benralizumab [38], are approved for severe asthma treatment. In addition, Tezepelumab, which blocks TSLP, an important regulator of airway remodeling, significantly reduced exacerbations, and improved lung function in patients with uncontrolled asthma [39]. Nevertheless, use of these drugs is effective against Th2-mediated eosinophilic response, but does not affect neutrophil infiltration. Therefore, the search for novel anti-cytokines strategies to control severe neutrophilic asthma is highly relevant.

TNF is a biomarker of severe asthma, and therapeutic agents that neutralize TNF were considered as anti-cytokine therapy. TNF orchestrates smooth muscle remodeling [14], immune cell recruitment [11,40], and maintenance of chronic inflammation in the airways. Several clinical studies have tested anti-TNF agents as therapeutics for patients with severe asthma. However, Golimumab, a human monoclonal antibody, demonstrated no therapeutic effects in uncontrolled asthma. Furthermore, patients on Golimumab experienced severe side effects, including life-threatening infections [17]. On the contrary, Etanercept and Infliximab significantly improved lung function in patients with moderate and severe asthma [41,42,43,44]. Of note, prolonged systemic anti-TNF therapy is associated with an increased risk of reactivation of chronic infections [18,19] such as *Mycobacterium tuberculosis* [45] and increased risk of neoplasia. In several studies, administration of TNF inhibitors also demonstrated encouraging results in mouse models of asthma [46,47,48]. In the present study, we confirmed that TNF inhibition abrogated Th2-mediated eosinophilia (Figure 1B and Figure 2A) in mice with HDM-induced asthma. However, the accumulation of Th1- and Th17-cells followed by neutrophilic infiltration into the lungs was not reduced in response to TNF blockade (Figure 1B, Figure 2C and Figure 3B). Thus, we hypothesized that TNF neutralization may be insufficient to fully control the progression of the disease. Since disease pathogenesis involves several cytokines with partially overlapping functions, we searched for other cytokines that could be linked to neutrophilia.

IL-6 is known to contribute to the induction and maintenance of chronic inflammation in respiratory tract [49]. IL-6 determines the type of adaptive immune response, directing effector CD4^+^ T cell fate [50]. On the one hand, IL-6 regulates Th2-cell differentiation by increasing Th2-associated cytokine production [51] and inhibiting Th1-cell expansion [52]. Additionally, IL-6 suppresses Treg cells and initiates Th17-cell differentiation [53]. The effects of IL-6 from different cellular sources may vary significantly. For example, mice with IL-6 deficiency in macrophages demonstrated reduced Th2-induced eosinophilic inflammation, while IL-6 deficiency in dendritic cells decreased Th17-mediated neutrophilic inflammation in the airways [54]. Furthermore, analysis of human bronchial tissue samples supported the involvement of IL-6 in airway remodeling during asthma [55]. Recent study evaluated clinical and immunological responses to Tocilizumab, a humanized anti-IL-6R monoclonal antibody, in severe asthma. Tocilizumab administration resulted in decreased Th2- and Th17-cell mediated inflammation, but did not affect peripheral eosinophilia [56]. However, a parallel clinical study found no evidence for Tocilizumab ability to prevent bronchoconstriction [57].

Targeting multiple cytokine pathways remains an attractive strategy to overcome excessive functions of pro-inflammatory cytokines. In particular, simultaneous blockade of IL-4Ra, a common part of the receptor complex of IL-4 and IL-13, with Dupilumab showed significant therapeutic effect and is approved for patients with severe eosinophilic asthma [8,9], despite poor efficacy of single anti-IL-4 and anti-IL-13 therapeutics [58,59,60,61]. Under inflammatory conditions TNF and IL-6 demonstrate some functional redundancy: for example, they both mediate inflammatory response during sepsis [62], and may act as immunometabolic transmitters [63,64,65]. On the other hand, due to distinct intracellular signaling of their cognate receptors, these cytokines may exhibit distinct functions or work synergistically to promote inflammation [66,67,68,69]. In the context of allergic airway inflammation, both TNF and IL-6 overproduction may trigger different inflammatory pathways implicated in asthma pathogenesis. We thus hypothesized that combined anti-IL-6/TNF treatment may be beneficial in the context of mixed granulocytic asthma and are reporting here that combined pharmacological inhibition of TNF and IL-6 indeed attenuated granulocyte infiltration in the airways (Figure 1B) and secretion of IgE in the BALF (Figure 1E) in mice exposed to HDM. Interestingly, TNF blockade reduced IgE production to the level observed in mice with combined IL-6/TNF inhibition (Figure 1E) indicating a potential role for TNF-mediated signaling in modulation of the B-cell compartment in asthma. Strikingly, mice under administration of anti-IL-6 antibodies showed decreased number of neutrophils, but not of eosinophils, whereas TNF blockade mostly suppressed accumulation of eosinophils (Figure 1B).

Recent advances indicate that asthma can no longer be considered solely as an IgE-mediated disease of the adaptive immune system. Instead, it should be viewed as result of a cross-talk between the innate and the adaptive immune responses [70]. Such cross-talk occurs when a dominating type of T-helper population, derived from naïve T-cells after allergen exposure, induces subsequent innate immune responses, including granulocyte infiltration and mast cell activation. Asthma has long been considered as a Th2-associated inflammatory disease, with IL-4, IL-5, and IL-13 as the key cytokines and eosinophilic infiltrates in the airways. However, the current paradigm suggests that the imbalance in other Th-cells, such as Th1-, Th17-, and Treg cells, is strongly implicated in asthma pathogenesis [71]. Moreover, the predominant type of T-helper cells may predict the severity of asthma: while asthma of moderate severity is characterized by Th2-cell activation, severe asthma is usually dependent on Th17/Th1-cell responses in the airways with subsequent neutrophilic infiltration [72]. Therefore, targeting key cytokines that drive a specific type of T-cell response is a promising strategy for personalized asthma therapy [3,26].

TNF mediates Th1 response and is produced by a wide spectrum of immune cells, such as macrophages, mast cells, and granulocytes. TNF participates in training macrophages to rapidly mediate Th2 response following repetitive allergen exposure [16]. On the other hand, IL-6 orchestrates Th17 cell differentiation and may enhance Th17-driven neutrophilic inflammation in the airways [27,54,73,74,75]. In the present study, we demonstrated that combined pharmacological inhibition of TNF and IL-6 prevents the accumulation of both Th2- and Th1/Th17-cells in the lungs (Figure 2A,C and Figure 3B). Administration of anti-IL-6 antibodies during asthma induction increased IL-13, IL-4, and IL-5 protein levels in the BALF (Figure 2B). These cytokines are well-known regulators of Th2-mediated eosinophilia; therefore IL-6 inhibition may enhance type 2 response in asthma. Interestingly, the effect of anti-IL-6 on Th2-associated cytokines was not observed in the serum (Figure S5), suggesting that the effect of anti-IL-6 is localized to the site of inflammation. Although administration of IL-6 inhibitor did not alter Th2 response to the allergen (Figure 2A), it resulted in reduced Th1- and Th17-cell accumulation in the lungs (Figure 2C and Figure 3B). On the other hand, TNF ablation did not reduce the Th1- and Th17- cells (Figure 2C and Figure 3B) nor *Il17a* relative expression levels in the lungs (Figure 3C). In line with this, several studies have established that TNF blockade may enhance IL-17A secretion and Th17-cell expansion [76]. Finally, despite systemic administration of cytokine inhibitors, systemic inflammatory response was not affected even under combined neutralization of TNF and IL-6 (Figures S3 and S5).

Pathological mechanisms during asthma induce pulmonary tissue destruction and injury. Airway remodeling is an abnormal process that is controlled by a cross-talk of different cell subsets, leading to epithelial disruption, increased production of extracellular matrix (ECM), airway smooth muscle (ASM) proliferation, fibroblast activation, and goblet-cell hyperplasia [77,78,79]. These modifications cause asthma-associated symptoms and lead to decreased respiratory function [80]. To further characterize the impact of IL-6/TNF inhibition in asthma we used a high concentration of HDM, administrated intratracheally, to induce more abundant inflammatory response and tissue remodeling in mice. Histological assessment as well as FACS analysis revealed reduced infiltration of immune cells into lung tissue in mice undergoing combined inhibition of TNF and IL-6 (Figure 4B). The observations were further corroborated by histological scoring of the inflammatory cell infiltration (Figure 4C) and PAS-staining, indicating a decline in the number of PAS-positive cells under simultaneous inhibition of TNF and IL-6 (Figure 4D). TGF-β, IL-4, IL-9, IL-13, IL-17, and vascular endothelial growth factor (VEGF) are the major mediators of airway remodeling [81]. TGF-β is involved in initiation and regulation of fibrotic tissue remodeling in asthma [82] and expression level of TGF-β correlates with asthma severity. TGF-β is known for its central role in epithelial barrier injury [83]; it also stimulates IL-6 production, that, in turn, enhances airway smooth muscle proliferation [84] and induces mucus overproduction [85]. In addition, TGF-β represents a crucial initiator of collagen production in lung tissue and its expression is elevated both in human and animal lung fibrotic tissues [86]. TGF-β initiates differentiation of fibroblasts [86]. Moreover, proliferation and trans-differentiation of fibroblasts to myofibroblasts depend on TNF- and IL-6-induced expression of TGF-β [30,31,87]. In this study, we found that simultaneous inhibition of TNF and IL-6 prevented the increase in *Tgfb1* expression, in contrast to what was observed following neutralization of either of these cytokines individually (Figure 4E). Importantly, TGF-β expression is not altered by corticosteroid treatment [88]. Thus, we propose that combined anti-IL-6/TNF administration may represent a novel approach to prevent TGF-β-induced airway remodeling. Further studies are required to investigate the efficacy of simultaneous inhibition of TNF and IL-6 on the activity of other asthma-related markers that are crucial for airway remodeling.

In general, conventional anti-cytokine therapy possesses some disadvantages such as systemic side effects. Owing to functional redundancy of cytokines, including their homeostatic functions, a single cytokine inhibition may be ineffective for asthma therapy. Inhibition of several important cytokines may exacerbate side effects. As shown here, combined anti-cytokine therapy may represent a better treatment strategy due to less severe side effects, as compared under monotherapy, and/or due to lower doses of systemic blockers [89] that may reduce the risk of complications. More selective approaches, such as cell-type restricted or local cytokine inhibition could also help reduce adverse effects [90,91].

**Figure 5:**
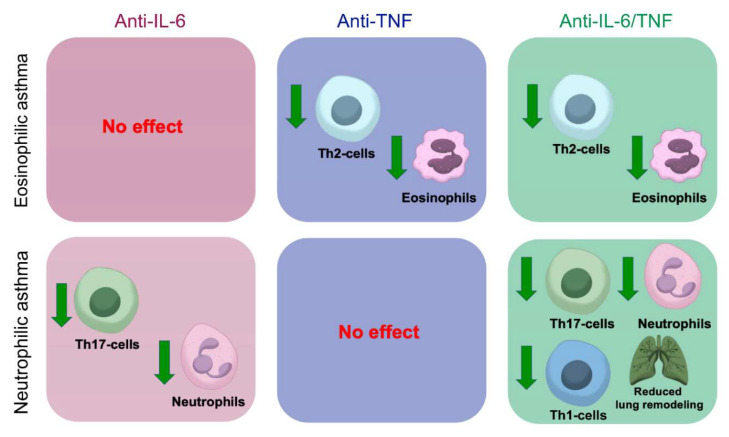
Combined anti-IL-6/TNF administration induces a complex anti-inflammatory effect in the context of severe asthma. Inhibition of IL-6 demonstrated decreased frequency of neutrophils, whereas TNF ablation predominantly suppressed eosinophilic infiltrate into the airways. Moreover, administration of IL-6 neutralizing antibodies prevented the accumulation of Th17- and Th1- cells in the airways, caused by pharmacological TNF ablation. Th2-associated eosinophilia is inhibited under TNF blockade and was not observed under anti-IL-6 administration. On the contrary, combined pharmacological inhibition of IL-6 and TNF had a complex effect consisting of reduction in both Th2-associated eosinophilia and Th1/Th17-mediated neutrophilia and prevention of tissue remodeling in the airways. Therefore, combined anti-IL-6/TNF therapy may be beneficial in inhibiting the key inflammatory responses in allergic airway inflammation and preventing the side effects of monotherapy.

Taken together, our results demonstrate a beneficial effect of combined anti-TNF/IL-6 administration including reduced Th2-associated eosinophilia and Th17/Th1-mediated neutrophilic infiltrate in the airways (Figure 5).

Moreover, simultaneous administration of IL-6 and TNF inhibitors more effectively protects the lungs from asthma-associated tissue remodeling. Our study provides support for a novel therapeutic strategy for severe asthma associated with mixed granulocyte inflammation.

## 4. Materials and Methods

Mice. Female BALB/c mice, 6–8 weeks of age (15–20 g). Mice were housed under the standard conditions of the Animal Breeding Facility, BIBCh, RAS (the Unique Research Unit Bio-Model of the IBCh, RAS; the Bioresource Collection—Collection of SPF-Laboratory Rodents for Fundamental, Biomedical, and Pharmacological Studies) accredited at the international level by AAALACi. The BALB/c mice were selected due to their high sensitivity to the development of allergic airway inflammation [92,93]. All animal experiments were approved by the local authorities IACUC committee of the Branch of Shemyakin–Ovchinnikov Institute of Bioorganic Chemistry, Russian Academy of Sciences (protocol no. 125, 29 December 2020) and performed according to institutional guidelines and the Scientific Council of the Engelhardt Institute of Molecular Biology, Russian Academy of Sciences.

Acute Asthma Protocol. Lyophilized house dust mite (HDM) extract from *Dermatophagoides pteronyssinus* (Greer Labs, Lenoir, NC, USA) was diluted with sterile saline at 100 μg per 50 μL. Batch XP B70D3A25 of HDM extract containing *Der p1* = 9.2 mg/g and *Der p2* = 11.2 mg/g was used. Mice were anesthetized by 3% isoflurane (Baxter, Deerfield, Massachusetts, USA) using the low-flow SomnoSuite anesthesia delivery system (Kent Scientific Corporation, Torrington, Connecticut, USA) prior to intranasal (i.n.) administration of HDM. To establish mixed asthma model with both eosinophilic and neutrophilic inflammation in response to HDM, previously published protocol was used [94] with minor modifications. Asthma was induced by daily administration (i.n.) of 20 µg HDM per mouse (at concentration of 1 µg/µL in PBS) for six days with the sensitization of 5 µg HDM one week prior to the main course (Figure 1A).

Severe Asthma Protocol. Mice were sensitized intratracheally (i.t.) with HDM extract on days 0 and 4 (100 μg). On days 14–17, mice were challenged i.t. with HDM (100 μg). Prior to i.t. administration of HDM extract, mice were anesthetized by intraperitoneal injection (i.p.) of the Zoletil/Xyla cocktail for anesthesia (Figure 4A).

Anti-cytokine Administration. Optimized doses of murine monoclonal antibody MP5-20F3 against IL-6 (5 μg/g of body weight) (BioXCell, Lebanon, NH, USA), TNF inhibitor Etanercept (Pfizer, UK) (10 μg/g of body weight), or monoclonal antibody XT3.11 against murine TNF (10 μg/g of body weight) (BioXCell, Lebanon, NH, USA) were injected i.p. prior to each intranasal immunization with HDM extract. Mice were randomized into four groups (*n* = 5–6 per group): HDM + saline, HDM + anti-IL-6, HDM + anti-TNF, and HDM + anti-IL-6 and anti-TNF (Figure 1A and Figure 4A).

Tissue Cell Isolation. Cell suspensions were prepared by mechanical dissociation of lymph nodes and spleens in phosphate buffered saline (PBS) supplemented with 2% fetal bovine serum (FBS) through a cell strainer (BD, Biosciences, Franklin Lakes, NJ, USA). Bronchoalveolar lavage fluid (BALF) was collected using a tracheal cannula with two washes of 0.8 mL of 5 mM EDTA in PBS followed by centrifugation for 7 min at 300 g and 4 °C. The resulting cell pellet was resuspended in RPMI 1640 culture medium supplemented with 2% FBS. To obtain airway infiltrating lymphocytes, lungs were isolated from ventricularly perfused mice and enzymatically digested in HEPES digestion cocktail (PBS contained 10 mM HEPES, 20,000 U/mL DNAse I; 100 mg/mL Collagenase D (Sigma, St. Louis, MO, USA)) by using the gentleMACS Octo Dissociator (Miltenyi Biotec, Germany) program “lung_01” (36 s, 165 rpr). Following incubation for 25 min at 37 °C, samples were further dissociated with gentleMACS Octo Dissociator program “lung_02” (37 s, 2079 rpr). The resulting cell suspension was centrifuged in a 40/80% Percoll gradient (GE Healthcare, Sweden) centrifugation for 25 min at 330 g and 4 °C without breaking. For further analysis, the resulting leukocyte pellet was resuspended in PBS supplemented with 2% FBS.

Flow Cytometry. Single cell suspensions obtained from isolated tissues or BALF were assessed for lymphocyte and myeloid cell populations. To limit nonspecific binding, Fc receptors were blocked with anti-CD16/CD32 (Invitrogen) for 20 min at 4 °C, followed by staining with antibodies against surface markers. Single cell suspensions were stained with fixable viability dye, anti-CD45, anti-SiglecF, anti-CD11c, anti-CD11b, anti-Ly6G, anti-TCRβ, and anti-CD4 antibodies (Table S1).

For intracellular staining of CD4^+^ T-cell cytokines, cells were stimulated with 50 ng/mL phorbol myristate acetate (PMA) (Sigma, St. Louis, MO, USA) in the presence of 500 ng/mL ionomycin and brefeldin A (Thermo Fisher Scientific, Waltham, MA, USA), incubated for 4 h at 37 °C and washed once with PBS supplemented with 2% FBS. After staining for surface markers, cells were fixed in permeabilization buffer with fixation/permeabilization kit (eBioscience), followed by intracellular staining with anti-IL-17A, anti-IL-13, anti-IFNγ, anti-TNF, anti-RORγt, anti-HELIOS, and anti-FoxP3 antibodies (Table S1). Gating strategies for identification of myeloid cells (Figure S1) and Th-cell subsets (Figure S2) are summarized in Supplementary Materials. The stained cells were analyzed using BD FACSCanto II cytometer and FlowJo v10 software.

ELISA and Multiplex Analysis. The supernatants from BALF and serum were collected for assessment of cytokine and IgE production using commercially available enzyme-linked immunosorbent assay (ELISA) kits (Invitrogen, Waltham, MA, USA) and a MILLIPLEX MAP Mouse Cytokine/Chemokine Magnetic Bead Panel-Premixed 32 Plex (MCYTMAG-70K-PX32, Merck, Darmstadt, Germany) according to the manufacturer’s protocol.

cDNA Preparation and RT-PCR. Lungs were homogenized in TRK Lysis Buffer (Omega Bio-tek, Norcross, GA, USA) using IKA T10 basic Ultra Turrax Homogenizer (IKA, Staufen, Germany) and RNA was extracted E.Z.N.A.^®^ Total RNA Kit I (Omega Bio-tek, Norcross, GA, USA), according to the manufacturer’s protocol. RNA was reverse-transcribed into cDNA using RevertAid First Strand cDNA Synthesis Kit (Thermo Fisher Scientific, Waltham, MA, USA) followed by quantitative real-time PCR. SYBR Select Master Mix (2X) (Applied Biosystems, Waltham, MA, USA) was used to amplify target genes with specific primers (Evrogen, Russia) (Table S2). Gene expression analysis was performed using a 7500 Real Time PCR System Amplificator (Applied Biosystems, Waltham, MA, USA).

Histology. Lung tissue samples were fixed in 4% paraformaldehyde (4% PFA) for 24 h and then embedded in paraffin. Following deparaffinization, 4 μm thick sections were stained with hematoxylin and periodic acid-Schiff (PAS) (Sigma, St. Louis, MO, USA) according to the manufacturer’s protocol. The level of inflammation and the abundance of airway goblet cells were evaluated using a previously reported semiquantitative scoring system with minor adaptations [95]. Briefly, inflammatory cell infiltration and peribronchial cell counts were determined by a 5-point grading system as follows: 0, normal; 1, few cells; 2, a ring of inflammatory cells one cell layer deep; 3, a ring of inflammatory cells of 2–4 cells deep; 4, a ring of inflammatory cells of more than 4 cells deep. To quantify airway goblet cells, the following 5-grading system was used: 0, <1% PAS-positive cells; 1, <25%; 2, 25–50%; 3, 50–75%; 4 >75%. The total score for 4 fields per each section was averaged between biological replicates in the groups.

Statistical Analysis. All figures presented include naïve group data. All experiments were independently performed at least three times. Statistical analyses were done using Prism 9 software (GraphPad Software, San-Diego, CA, USA). One-way ANOVA test was used; *p* < 0.05 was considered statistically significant.

## 5. Conclusions

In present study, we demonstrated that combined pharmacological inhibition of IL-6 and TNF had a complex effect manifested in reduction in both Th2-associated eosinophilia and Th1/Th17-mediated neutrophilia in HDM-induced asthma that was not observed under neutralization of these cytokines individually. Our results highlight the importance of personalized approach in selecting the adequate therapy for complex heterogenic diseases such as asthma.

## Data Availability

Data supporting the reported results are available on request from the corresponding author.

## Supplementary Materials

The following supporting information can be downloaded at: https://www.mdpi.com/article/10.3390/ijms23073521/s1.
