# Supplementary material for: Therapeutic Potential of Combining IL-6 and TNF Blockade in a Mouse Model of Allergic Asthma
Source: Int J Mol Sci. 2022 Mar 24;23(7):3521. doi: 10.3390/ijms23073521 (PMC8998171; doi:10.3390/ijms23073521)
Supplement: Supplementary file 1 [file ijms-23-03521-s001.zip › Supplementary materials _update-1 (1).pdf]

Therapeutic potential of combining IL-6 and TNF blockade in a mouse model of allergic asthma

Olga A. Namakanova, Ekaterina A. Gorshkova, Ruslan V. Zvartsev, Sergei A. Nedospasov, Marina S. Drutskay and Ekaterina O. Gubernatorova

Table S1

Table S1. Antibodies for flow cytometry.

Antigen	Fluorochrome	Clone	Company
Fixable Viability Dye	eFluor-780		eBioscience
CD45	PerCP-Cy7.5	30-F11	Biolegend
TCRb	PerCP-Cy7.5	H57-597	eBioscience
CD4	bv510	RM4-5	Biolegend
CD11c	APC	N418	eBioscience
CD11b	PE-Cy7	M1/70	Biolegend
SiglecF	PE	E50-2440	BD Pharmingen
Ly6G	FITC	1A8	Biolegend
IL-13	Alexa Fluor 433	eBio13A	eBioscience
IL-17A	PB	eBio17B7	eBioscience
TNF	PE	MP6-XT22	eBioscience
IFNγ	FITC	XMG1.2	eBioscience
RORγt	APC	B2D	eBioscience
HELIOS	FITC	22F6	Biolegend
FoxP3	APC	FJK-16s	eBioscience

Table S2

Table S2. Nucleotide sequences of primers used.

Gene name	Sequence	
	Forward	Reverse
Actb	CTCCTGAGCGCAAGTACTCTGTG	TAAAACGCAGCTCAGTAACAGTCC
Tgfb1	CAACAATTCCTGGCGTTACCT	GGCTGATCCCGTTGATTTCC
Il17a	GGACTCTCCACCGCAATGA	GGCACTGAGCTTCCCAGATC
Ifng	TCAAGTGGCATAGATGTGGAAGAA	TGGCTCTGCAGGATTTTCATG
Muc5ac	AGAATATCTTTCAGGACCCCTGCT	ACACCAGTGCTGAGCATACTTTT
Muc5b	TCCTGCTCTGGAATATCCAAG	GCCTCGGGGAGCTTGCCTGCC
Areg	ACCATAAGCGAAATGCCTTCTG	CTTAATCACCTGTTCAACTCTGACTG
Col1a1	ACGCCATCAAGGTCTACTG	GTACTCGAACGGGAATCCA
Il5	AGCACAGTGGTGAAAGAGACCTT	TCCAATGCATAGCTGGTGATTT
Il4	GGTCTCAACCCCCAGCTAGT	GCCGATGATCTCTCTCAAGTGAT
Il13	CCTGGCTCTTGCTTGCCTT	GGTCTTGTGTGATGTTGCTCA
Il10	CCAGTTTTACCTGGTAGAAGTGATG	TGTCTAGGTCCTGGAGTCCAGCAGACTCAA
Il33	TGCTCAATGTGTCAACAGACG	TCCTTGCTTGGCAGTATCCA

Figure S1

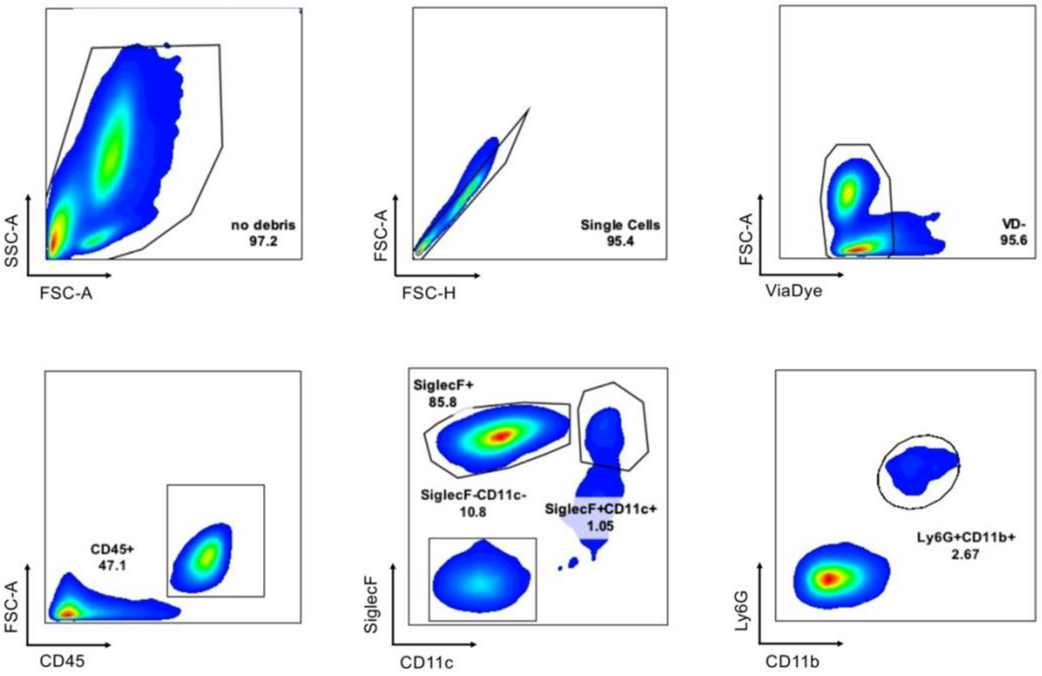

Figure S1. Flow cytometry gating strategy for identification of granulocytes in the BALF. Eosinophils and alveolar macrophages are indicated as ViaDye$^-$CD45$^+$SiglecF$^+$CD11c$^-$ and ViaDye$^-$ CD45$^+$ SiglecF$^+$ CD11c$^+$, respectively. Neutrophils Ly6G$^+$CD11b$^+$ were gated on SiglecF$^-$CD11c$^-$ cells. Frequencies (%) of SiglecF$^+$CD11c$^-$, Ly6G$^+$CD11b$^+$ and SiglecF$^+$CD11c$^+$ were counted from CD45$^+$ cells.

Figure S2

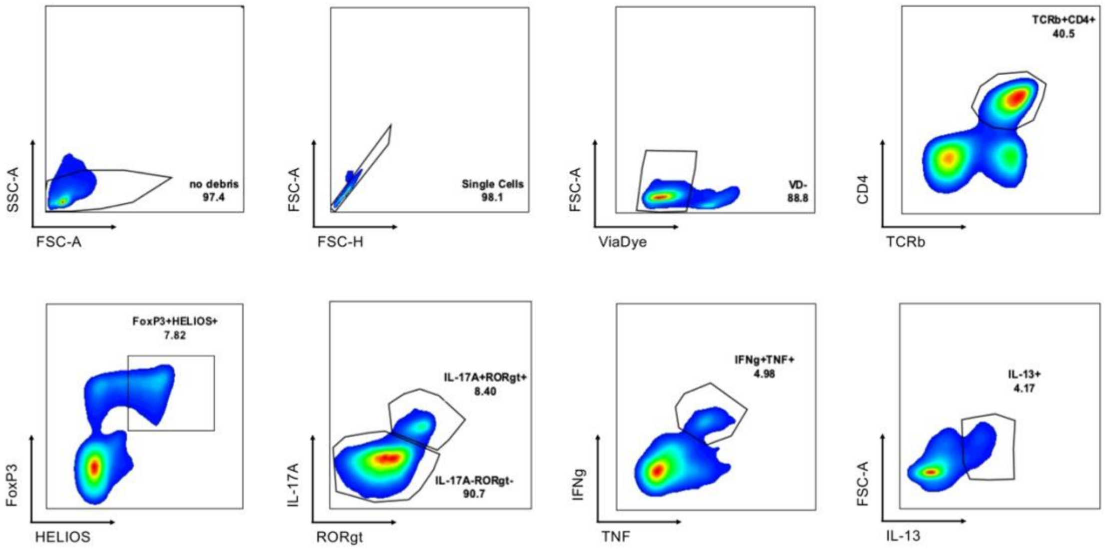

Figure S2. Representative flow cytometry gating strategy for identification of Th-cell subsets in the lungs and on the periphery. Activated Th-cells with PMA and ionomycin were identified as ViaDye$^-$ TCRb$^+$ CD4$^+$ cells. FoxP3$^+$ HELIOS$^+$, IL-17A$^+$ RORγt$^+$, TNF$^+$ IFNγ^+ and IL-13$^+$ Th-cells were gated on TCRb$^+$ CD4$^+$ cells, respectively.

Figure S3

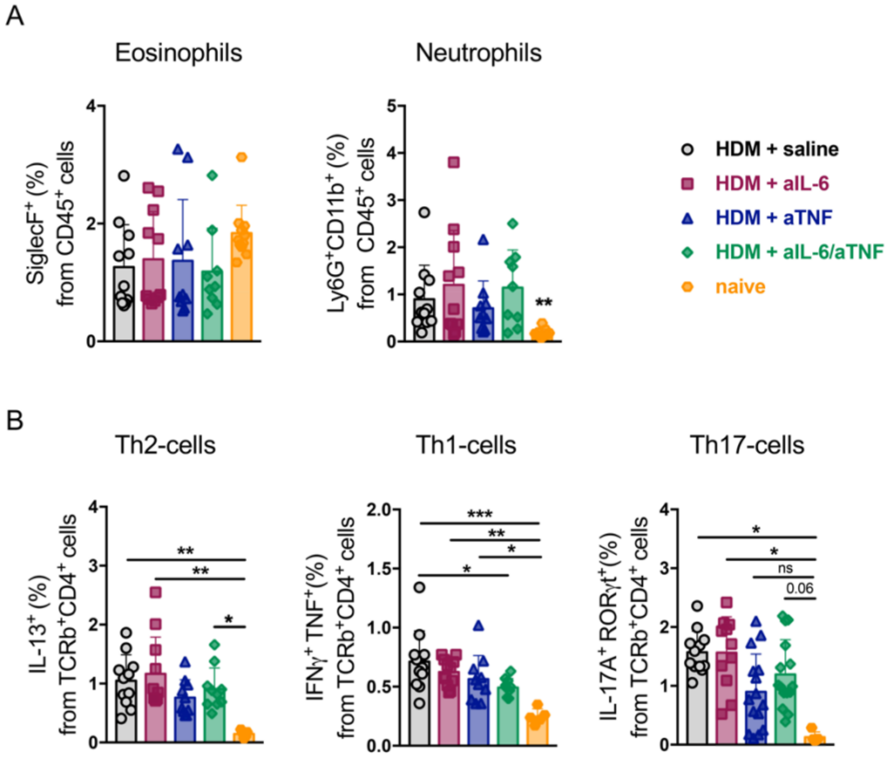

Figure S3. Analysis of myeloid cells and Th-lymphocytes in the spleen from mice under IL-6 and TNF inhibition and saline as a control. (A) The frequency (%) of eosinophils (Siglec-F$^+$ CD11c$^-$) and neutrophils (Ly6G$^+$ CD11b$^+$) gated on CD45$^+$ live cells. (B) Representative frequencies (%) of Th2-cells (IL-13$^+$), Th1-cells (TNF$^+$ IFNγ^+) and Th17-cells (IL-17A$^+$ RORγt$^+$) gated on TCRb$^+$ CD4$^+$ live cells. Data represent mean ± SD, 10-12 mice per group with each point representing a single mouse. * $p < 0.05$; ** $p < 0.01$; *** $p < 0.001$; ns, nonsignificant (one-way ANOVA test was used).

Figure S4

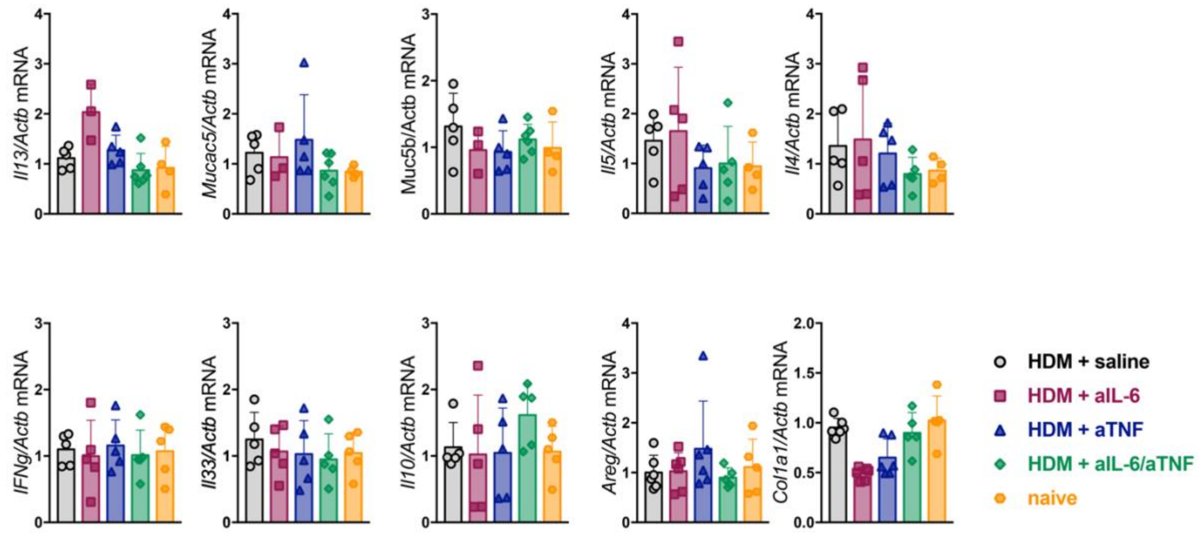

Figure S4. Quantitative RT-PCR analysis of asthma-associated genes. Relative expression of *Il13*, *Muc5ac*, *Muc5b*, *Il5*, *Il4*, *Ifng*, *Il33*, *Il10*, *Areg* and *Col1a1* genes were normalized to *Actb* in the lungs. Data are representative of 2 independent experiments with five mice per group in each experiment; mean ± SD. * $p < 0.05$ (one-way ANOVA test was used).

Figure S5

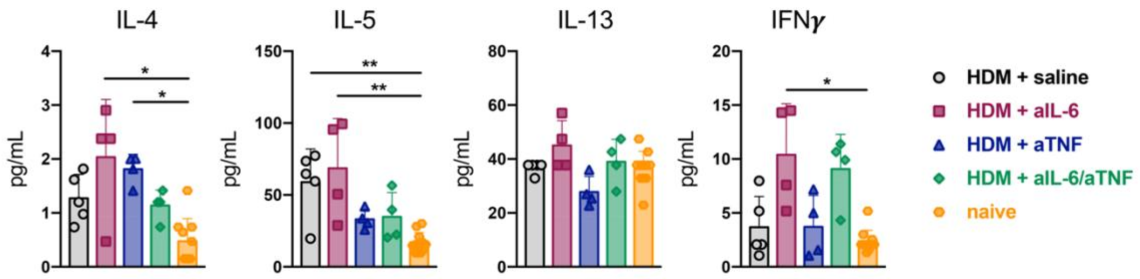

Figure S5. Quantification of cytokine production by multiplex analysis. Cytokine levels (pg/mL) in mouse serum were measured using a multiplex microbead-based immunoassay. Data are representative of 2 independent experiments; mean ± SD, 3-5 mice per group. * $p < 0.05$; ** $p < 0.01$ (one-way ANOVA test was used).
